# *Mycoplasma agalactiae* ST35: a new sequence type with a minimal accessory genome primarily affecting goats

**DOI:** 10.1186/s12917-021-03128-w

**Published:** 2022-01-11

**Authors:** George Filioussis, Georgios Bramis, Evanthia Petridou, Nektarios D. Giadinis, Laurent-Xavier Nouvel, Christine Citti, Joachim Frey

**Affiliations:** 1 Laboratory of Microbiology and Infectious Diseases, Faculty of Veterinary Medicine, School of Health Science, Aristotle University of Thessaloniki, University Campus, 54124 Thessaloniki, Greece; 2 Laboratory of Animal Husbandry, Faculty of Veterinary Medicine, School of Health Science, Aristotle University of Thessaloniki, University Campus, 54124 Thessaloniki, Greece; 3 Clinic of Farm Animals, Faculty of Veterinary Medicine, School of Health Science, Aristotle University of Thessaloniki, St. Voutyra 11, 54627 Thessaloniki, Greece; 4 IHAP, Université de Toulouse, INRAE, ENVT, Toulouse, France; 5 Vetsuisse Faculty, University of Bern, Laenggasstrasse 120, 3001 Bern, Switzerland

**Keywords:** *Mycoplasma agalactiae*, Sequence type 35, Goats, Full genome, Contagious agalactia

## Abstract

**Background:**

*Mycoplasma agalactiae*, causing agent of contagious agalactia, infects domestic small ruminants such as sheep and goats but also wild Caprinae. *M. agalactiae* is highly contagious and transmitted through oral, respiratory, and mammary routes spreading rapidly in an infected herd.

**Results:**

In an outbreak of contagious agalactia in a mixed herd of sheep and goats, 80% of the goats were affected displaying swollen udders and loss of milk production but no other symptom such as kerato-conjunctivitis, arthritis or pulmonary distress commonly associated to contagious agalactia. Surprisingly, none of the sheep grazing on a common pasture and belonging to the same farm as the goats were affected. Whole genome sequencing and analysis of *M. agalactiae* strain GrTh01 isolated from the outbreak, revealed a previously unknown sequence type, ST35, and a particularly small, genome size of 841′635 bp when compared to others available in public databases. Overall, GrTh01 displayed a reduced accessory genome, with repertoires of gene families encoding variable surface proteins involved in host-adhesion and variable antigenicity being scaled down. GrTh01 was also deprived of Integrative Conjugative Element or prophage, and had a single IS element, suggesting that GrTh01 has a limited capacity to adapt and evolve.

**Conclusions:**

The lack of most of the variable antigens and the Integrative Conjugative Element, both major virulence- and host specificity factors of a *M. agalactiae* strain isolated from an outbreak affecting particularly goats, indicates the implication of these factors in host specificity. Whole genome sequencing and full assembly of bacterial pathogens provides a most valuable tool for epidemiological and virulence studies of *M. agalactiae* without experimental infections.

## Introduction

*Mycoplasma agalactiae* is an important pathogen causing contagious agalactia in small ruminants such as sheep, goats and wild Caprinae. Contagious agalactia is a notifiable disease listed by the World Organisation for Animal Health (OIE) due to the important economic losses to the dairy industry and to small ruminants holders [1]. The contagious agalactia caused by *M. agalactiae* includes clinical symptoms of mastitis, arthritis and kerato-conjunctivitis, but *M. agalactiae* infections occasionally also cause abortions, pneumonia and septicaemia in goat and sheep [2, 3]. *M. agalactiae* is highly contagious and transmitted through oral, respiratory, and mammary routes. Since the end of the nineteenth century, the disease had been reported to be enzootic in many parts of the world [1, 4, 5]. Outbreaks of *M. agalactiae* infections occur notably in the Mediterranean basin, India, Australia, Turkey, Iran, Mongolia, Nigeria, Senegal and Iraq [4]. The spread of contagious agalactia within a population is multifactorial including inadequate herding practices, inefficiency of antimicrobial therapies, and adoption of very few prophylactic measures. In addition, the high potential of *M. agalactiae* to persist in the host and to some extent in the environment often renders difficult to eradicate the infections. The disease is mostly reported from sheep where it causes highest economic losses mainly due to high morbidity rather than high mortality resulting in losses of milk production throughout the world, but goats are equally affected by *M. agalactiae* infections [4]. *Mycoplasma agalactiae* possess a family of related surface lipoproteins, named variable protein of *M.agalactiae* (Vpma) that are prone to high-frequency variation in expression and structure thus modulating the mycoplasma surface structure and accessibility to immune components of the host. This provides *M. agalactiae* the capability to rapidly adapt to various environmental changes and to evade the host’s immune defence. Phase and size variations have been shown to occur in vitro with frequencies estimated at 10E-2 to 10E-5 events/cell/generation [6]. *M. agalactiae*, type strain PG2 which was used for most molecular studies, carries a genetic locus containing a cluster of 6 *vpma* genes (*vpmaU* though Z), while other *M. agalactiae* strains such as strain 5632 show up to 18 different *vpma* genes occurring as two clusters on two different loci of the chromosome. In a given cell, only one *vpma* gene per locus is expressed, while in the overall population the different *Vpma* antigens occur at different rates [6–8]. The intra-clonal variation is due to the presence of a unique promoter at the *vpma* loci that is alternatingly placed by a DNA recombination mechanism in front of silent *vpma* genes, resulting in ON and OFF switching of the various Vpma expression. Vpma phase variation allows *M. agalactiae* to survive in immuno-competent hosts and to establish persistent infections and cause debilitating disease [9, 10]. The phase variation, which is important for the host immune evasion and the in-host survival of the pathogen is controlled to a major extent by the Xer1 recombinase, also named DNA invertase Mar, that is encoded by the *vpma* loci [11, 12]. Recently a novel role of Vpmas as major adhesins of *M. agalactiae* mediating differential adhesion and invasion of host cells has been unravelled by the use of phase locked mutants expressing specific well-characterized Vpma lipoproteins [13].

In order to better understand the origin of *M. agalactiae* infections and their mode of propagation within and among herds with the aim of controlling the disease, molecular epidemiological tools including variable-number tandem repeat (VNTR) and multi-locus sequence typing (MLST) have been developed. They have been used successfully to trace *M. agalactiae* outbreaks and emergence or re-emergence after eradication campaigns [2, 5, 14–17]. While certain European countries such as Spain seem to have endemic clones, in particular ST5, others such as Greece have to deal with several different clones including ST2, ST4, ST6, and ST8 [5, 17–19]. It was also shown that *M. agalactiae* strains circulating in the ibex (*Capra ibex*) population in the French Alps were highly interrelated and most likely originated from a single parental clone that has also spread to chamois (*Rupicapra rupicapra*), another wild ungulate species in the same geographical area [2]. The clone contains some genetic loci closely related to *Mycoplasma conjunctivae*, a pathogen that is frequently found in wild ungulates with infectious kerato-conjunctivitis (IKC) [2, 20–22]. However, the molecular basis of the potential specificity or predilection of the clone to the wild Caprinae ibex and chamois is still unknown. The current manuscript describes a particular strain of *M. agalactiae* of a previously undetected sequence type, ST35 that caused an uncommon outbreak of contagious agalactia affecting specifically goats. The outbreak strain had a particularly small genome size and lacked several characteristic genes involved in host adhesion and variable antigenicity.

## Materials and methods

### Observation farm

The observation farm was located in Aridea (northwestern Greece), a lowland at 120 m above sea level. The farm harboured a mixed herd of 850 Lacaune milking sheep (*Ovis aries*) and 320 Anglo-nubian milking goats (*Capra aegagrus hircus*) of the age of 1-12 years. About one third (106) of the animals were aged 15 – 17 months and were the first time in the milking season. Sheep and goats were kept in separate barns but shared meadows and were milked at the same milking place. Generally, all adult animals were vaccinated twice a year against contagious agalactia with a commercial vaccine (Agalax Uno; Laboratorios Syva, Leon, Spain) based on inactivated bacteria of *Mycoplasma agalactiae*. However, the year of the observed outbreak, no vaccination was recorded for both sheep and goats.

### Clinical examinations

A veterinarian conducted a general clinical routine examination inspecting eye, ears, mouth, body, skin, hair, rectum vagina, udder, legs, hooves and records of appetence as recommended by Nagy and Pugh [23] giving special attention to udders as described for previous studies [24].

### Bacteriological diagnosis

Composite milk samples (CMS) were collected from seven randomly selected goats that suffered from clinical mastitis, before any treatment was administered. Briefly, following proper disinfection of the teats and the withdrawal of the initial 3 to 4 squirts of milk, CMS (ca. 30 mL each), were collected in sterile falcon tubes. The samples were refrigerated and submitted in insulated boxes containing coolants to the laboratory of microbiology and infectious diseases at the Faculty of Veterinary Medicine of Aristotle University, Thessaloniki within 12 h of collection. Aliquots of 10 μL milk were cultured on Columbia Agar supplemented with 5% sheep blood (Oxoid Ltd., Cambridge, United Kingdom), MacConkey Agar (Biolife Italiana S.r.l., Milan, Italy) and Sabouraud Dextrose Agar (Oxoid Ltd., Cambridge, United Kingdom) aerobically and in an atmosphere that contained 5% CO_2_ at 37^o^ C.

### Detection of mycoplasmas, isolation, culture, extraction of genomic DNA, genome sequencing and annotation

In order to detect the presence of mycoplasmas 0.5 mL of each CMS was inoculated in 5 mL of Heyflicks broth and incubated at 37 °C under an atmosphere of 5% CO_2_. Additionally 50 μL milk volume was cultured on Heyflicks agar and incubated at 37 °C under an atmosphere of 5% CO_2_. Identification of *Mycoplasma* colonies was performed using quick lysis DNA extraction and polymerase chain reaction (PCR) using primers, specific for *M. agalactiae* and the *Mycoplasma mycoides* cluster [25, 26].

A strain (GrTh01) of *M. agalactiae* was obtained from one of the *M. agalactiae* isolates by filter cloning through a 0.2 μm filter and sub-culture of a single colony as described [27]. For preparing genomic DNA the strain was grown in Heyflicks broth under aerobic atmosphere for 72 h. *Mycoplasma* DNA was extracted using the Guanidine thiocyanate extraction method [28] with two subsequent phenol extractions and two ethanol precipitations.

The genome sequence of *M. agalactiae* strain GrTh01 was generated using a combination of Pacific Biosciences RSII and Illumina HiSeq sequencing. After sequencing and circularization using Pacific Biosciences (PacBio) RSII (Swiss Institute of Bioinformatics, Lausanne, Switzerland), the sequence was corrected with Illumina HiSeq data for the substitutions due to sequencing errors and for the frameshifts, using the Celera Assembler (CA) PacBio correction module PBcR (version 7.0) [29]. Illumina reads were obtained using the Nextera XT DNA protocol for the library preparation and the V3 chemistry to produce 2 × 150 bp paired-end reads that resulted in 9.1 million read pairs (GATC Biotech, Konstanz, Germany). The resulting full genome was annotated using the Prokka pipeline [30] and the NCBI Prokaryotic Genome Annotation Pipeline PGAP https://www.ncbi.nlm.nih.gov/genome/annotation_prok/ and deposited at NIH genetic sequence database GenBank. The GenBank/EMBL accession number of the full genome sequence of *M. agalactiae* strain GrTh01 is CP039447. Detailed analysis of Vpma loci, potential virulence genes and comparisons of genomes of *M. agalactiae* strains were performed using the software package Geneious® software package 9.1.8 (Biomatters Ltd., Auckland 1141, New Zealand), Virulencefinder https://cge.cbs.dtu.dk/services/VirulenceFinder/ [31] and Artemis [32]. Multi locus sequence typing (MLST) was performed using the *M. agalactiae* MLST Databases (https://pubmlst.org/magalactiae/) extracting the gene-loci of *dnaA*, *gltX*, *gyrB*, *metS* and *tufA* from the genome sequence [33].

## Results

### Outbreak description

Between April and June 2016, 87 out of 106 young milking goats of the age of 15 to 17 months that were their first milking season showed a general malaise, absence of appetite, and signs of clinical mastitis. In most of the cases, both the right and the left udder were hot, swollen and tender while the milk became discolored and granular. No signs of respiratory complications, arthritis or keratitis were detected. Furthermore, the sheep population of the same farm remained unaffected, showing no clinical signs of contagious agalactia. The study of the medical records of the farm showed that, for an undefined reason, the whole farm (sheep and goats) was not vaccinated against contagious agalactia during the year preceding the outbreak. After the outbreak the goats of the farm were culled.

### Bacteriological analyses

Milk samples of 7 of the 87 affected goats were analysed bacteriologically of which all 7 showed *M. agalactiae* by culture and subsequent species differentiation using PCR. No other bacterial species were diagnosed from the 7 milk samples. One *M. agalactiae* isolate was twice filter cloned, and the strain isolated named GrTh01 was used for full genome analysis.

### Whole genome sequencing and analysis of strain GrTh01

The whole genome sequence (WGS) of *M. agalactiae* strain GrTh01was obtained by combining PacBio and Illumina sequencing and revealed a circular chromosome of 841′635 bp (GenBank full Genome accesion nr. CP039447). The Illumina HiSeq coverage was of 1895 x. As for the other *M. agalactiae* strains so far sequenced, no plasmid was identified in GrTh01. Annotation of the genome using the NCBI Prokaryotic Genome Annotation Pipeline PGAP revealed a total of 767 genes of which 623 encode proteins. Strain GrTh01 contains two full sets of rRNAs (5S, 16S, 23S), 34 tRNAs and 3 non coding RNAs (Table 1).

**Table 1:** General properties of *M. agalactiae* circularized genomes and corresponding strains. Note the full genome sequence of *M. agalactiae*, strain JF4428 is also available but not included in this table as JF4428 is a sample of strain PG2 that was propagated in vitro as internal diagnostic control for several decencies

	PG2	5632	GrTh01
Date of isolation	1952	< 1991	2017
Country	Spain	Spain	Greece
Source	unknown	articulation	milk
Host	caprine	caprine	caprine
Genome size (bp)	877,438	1,006,702	841,635
G + C (%)	29.70	29.60	29.80
Total number of CDS	657	782	626
rRNAs sets	2	2	2
tRNAs	34	34	34
GenBank accession number	CU179680	FP671138	CP039447
ICE number	0 (+ 2 vestigial)	3 (+ 2 vestigial)	0 (+ 2 vestigial)
Transposases	1 (+ 2 pseudogenes)	15 (+ 2 pseudogenes)	0 (+ 2 pseudogenes)

The GrTh01 MLST analysis revealed a new sequence type, namely ST35, having the following profile: *dnaA*: 21; *gltX*: 1; *gyrB*: 1, *metS*: 2; *tufA*: 1. ST35 stands apart because of its novel *dnaA* allele, which sequence was verified by additional Sanger sequencing using primers recommended by Mc Auliffe [33]. The MLST analyses also indicated that the ST35 GrTh01 strain clustered together with several strains isolated from Greece, with *dnaA* being the only discriminating allele and from the PG2 type strain isolated in Spain, with two discriminating alleles (Table 2). The *dnaA* allele in strain GrTh01 leads to a T/S change at a.a. position 91. While strain GrTh01 is currently the only strain among *M. agalactiae* and the phylogenetically closely related *Mycoplasma bovis*, the mutation is in a serine-rich area of DnaA and a serine residue is found at the analogous site of DnaA of *Mycoplasma simbae* (GenBank accession nr. WP 051630113).

**Table 2:** Allelic profiles and sequence types (ST) of *M. agalactiae* type strain PG2 and field strains isolated from Greece. Data taken from https://pubmlst.org/magalactiae/

Strain	Origin	Year of isolation	Allelic profile					ST
			dnaA	gltX	gyrB	metS	tufA	
NCTC 10123 (PG2)	USA	1983	1	1	1	1	1	1
**GrTh01**	**Greece**	**2016**	**21**	**1**	**1**	**2**	**1**	**35**
222F03	Greece	2003	1	1	1	2	1	2
233F03	Greece	2003	1	1	1	2	1	2
240F03	Greece	2003	1	1	1	2	1	2
235F03	Greece	2003	1	1	2	2	1	4
234F03	Greece	2003	1	1	2	4	1	6
224F03	Greece	2003	1	2	2	3	1	8
225F03	Greece	2003	1	2	2	3	1	8
227F03	Greece	2003	1	3	1	2	1	9

**Fig. 1:**
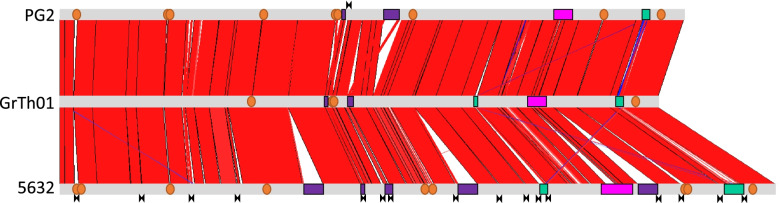
Genome comparison of *M. agalactiae* strains PG2 (Type strain) (upper), GrTh01 (mddle) and 5632 (lower) whose full circularized genomes are available. Orange circles: *bpsA*. Green, *vpma* loci. Red blocks show homologous genome stretches. Magneta: *spma* locus. Purple blocks: ICE or ICE vestiges (smaller blocks).  : IS elements

Of the currently publicly available *M. agalactiae* genomes, the GrTh01 genome is the smallest showing ca. 36 and 165 Kbp less than the two representative *M. agalactiae* strains with circularized genome, namely PG2 and 5632 [7, 34] (Table 1). More specifically, comparison of GrTh01 with these two strains revealed that GrTh01 lacks the 23-kb Integrative Conjugative Element (ICE) found in 5632 in three copies and the 20-kb ICE vestige of PG2 [34, 35] (Fig. 1). Interestingly, GrTh01 displayed 2 small ICE vestiges of ca 2.4 and 6.0 kb that are composed of only few CDSs and pseudogenes. The smallest is found in the three strains at the same locus, while the largest is only found in 5632 and GrTh01 (Fig. 1). Of note, PG2 and GrTh01 are deprived of IS elements, a situation that contrasts with 5632 having 15 belonging to the IS30 family [34].

Other smaller deletions, detected in GrTh01 when compared to PG2 or 5632, mostly affect gene families encoding surface proteins. More specifically, the repertoire of the Bacteroides like surface protein A (BspA) gene family is the smallest with 4 entire and 4 pseudo-genes in GrTh01 for 9 and 12 full copies in PG2 and 5632, respectively [34]. Of note, this family was previously described and annotated as *drp* genes by Nouvel et al. [34] or as PARCEL in *M. bovis* by Wise et al. [36] and were characterized by the presence of the motif DUF285, also present in BspA. In this study, we refer to all this CDS as *bspA*. The function of the proteins carrying a DUF285 domain is not known but these occurred across a large number of mycoplasma species and bacteria. In strain GrTh01, the *spma* locus that encodes a family of surface lipoprotein with unknown function is 13,475 bp and similar to PG2. Finally, as shown in Fig. 1, strain GrTh01 displayed two *vpma* loci as in 5632, a situation that contrast with the single locus of PG2 [34].

**Fig. 2:**
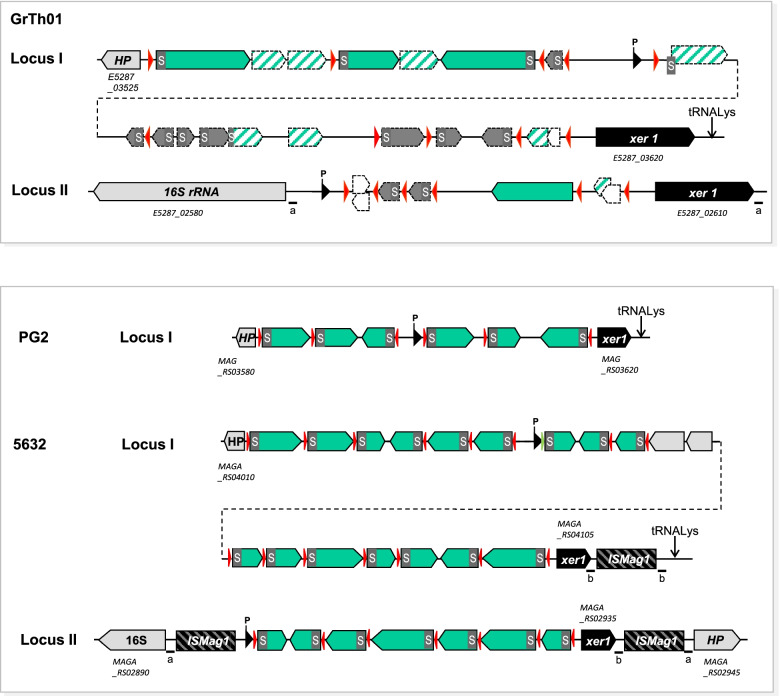
Genetic loci of *vpma* genes in *M. agalactiae* strain GrTh01. Arrowhead-Boxes represent open reading frames (ORF). Green arrowheads: *vpma*. Hatched green arrowheads partial or degenerated *vpma* genes. Black arrowheads: promoter sequences. Red arrowheads: 21 nt ‘vis’ motif encoding the Xer1 recombinase recognition site. Grey boxes labelled with ‘S’: signal sequences. HP: hypothetical protein

One striking feature of the GrTh01 loci is that they are highly degenerated, with only four genes encoding a signal peptide, a conserved N-terminal and a *vpma* block of amino acids (Fig. 2). Multiple copies of sequences encoding either a signal peptide and a few amino acids or a Vpma block without a signal sequence were identified on both loci (Fig. 2). These loci also contained multiple “*vis”* motifs, a highly conserved sequence of 21 nt that is recognized by the Xer recombinase, which one is responsible for DNA rearrangements [37] (Fig. 2). As in 5632, each of the two *vpma* loci contains a highly conserved *xer1* gene (100% nucleotide identity in between the two copies). In PG2 and 5632, each *vpma* locus possesses a single conserved promoter placed upstream of the expressed *vpma* gene, so that a maximum of 1 and 2 Vpma are expressed in PG2 and 5632, respectively. Here, the conserved promotor sequence was detected, but no entire *vpma* gene was found directly behind raising the question of whether the sequenced GrTh01 strain expressed a Vpma or whether another promoter, yet unidentified, is used by this strain for Vpma expression at the surface of the cell. Remarkably, GrTh01 locus II is flanked by two short direct repeats also found in 5632 in which they were generated upon IS integration (Fig. 2). Full identical copies including promoter and ribosome binding site were found in both strains PG2 and GrTh01 for the virulence genes *nifS* – *nifU* [38], the gene encoding the adhesin P40 (Fleury et al., 2002), and the lipoprotein P80 [39] that is involved in neutrophil extracellular trap (NET) formation [40].

## Discussion

A particular strain of *M. agalactiae*, strain GrTh01, was isolated from an outbreak of caprine contagious agalactia that was restricted to *Carpinae* and did not affect sheep that were grazing on the same meadows and kept in the same farm as the affected goats. Also rather uncommon was the profile disease symptoms that was restricted to mastitis and did not reveal arthritis, conjunctivitis or respiratory distress. Full genome analysis revealed that the chromosome of GrTh01 was significantly shorter than type strain PG2 and other fully sequenced *M. agalactiae* strains. Type strain of *M. agalactiae* PG2 was originally isolated from a goat but shown in several infection experiments to efficiently infect sheep [38]. There are no differences between PG2 and GrTh01 in the essential housekeeping genes, the virulence associated genes *nifS* and *nifU* that are required for host invasion, protein P40 involved in cell adhesion or the lipoprotein P80 involved in NET formation. However, strain GrTh01 lacks a locus of 20 kbp including conjugal transfer genes such as *traE* and DDE transposase and genes belonging to a potential type IV secretion system whose function in *M. agalactiae* is unknown. Furthermore, GrTh01 fully lacks genes encoding transposases such as the IS*30* transposase gene that is present in type strain PG2. Most interestingly, strain GrTh01 shows deletions in two groups of genes that both are potentially involved in adhesion: i) genes encoding the Bacteroides-like surface protein A (BspA) and ii) the *vpma* genes of the variable proteins of *M. agalactiae*. All currently sequenced strains of *M. agalactiae* possess several gene copies coding BspA family leucine-rich repeat surface proteins. While strain PG2 contains 9 full copies and 3 truncated *bspA* genes, and strain 5632 has 12 full, and one truncated copy, strain GrTh01 only contains 4 full copies and 4 truncated versions of *bspA*. The BspA protein includes a domain that seems to have regulatory function by suppressing the σ54 dependent transcription and was found to have adhesin functions. In *Tannerella forsythia* the BspA-like protein is essential in pathogenesis [41]. In *Entamoeba histolytica* the BspA-like protein binds to human TNF receptor 1 (TNFR1) and is essential for invasion of the human colonic mucosa possibly via a fibronectin/fibrinogen integrin binding process [42]. Hence, the various BspA proteins in *M. agalactiae* can be assumed to play a role in adhesion in host cell adhesion and tropism.

The *vpma* loci of *M. agalactiae* are considered as pathogenicity island-like loci possessing key features of pathogenicity islands such as the *tRNA-lys* gene, the invertase-recombinase *xer1* and the virulence associated *vpma* genes [37]. Furthermore, the Vpma proteins have been shown to be involved in virulence features such as immune evasion and immunomodulation and colonization of *M. agalactiae* in particular and *Mycoplasmas* in general [2, 6, 8, 43]. More recently, their involvement in differential cell adhesion and invasion was shown by the analysis of phase locked mutants that steadily express single well-characterized Vpma antigens [11, 13]. Co-infections of sheep with phase locked mutants stably expressing VpmaY and VpmaU revealed a dominance in the host of VpmaY expressors in particular during early stages of conjunctival and intra-mammary infections compared to VpmaU expressers, indicating differential roles of the antigenic variants in infection. Their data also revealed the importance of antigenic variation for survival and persistence inside the immunocompetent host as Xer1 independent antigenic variation was detected in the sheep infection model with Xer1 phase locked mutants [11]. While the surface antigenic variation caused by the Vpma proteins was mostly shown to be implicated as important mechanisms for better survival and persistence of *M. agalactiae* inside the host, a recent study using phase-locked mutants expressing the various Vpmas of strain PG2 revealed the role of Vpmas in adhesion [13]. Using sheep primary mammary epithelial cells and mammary stromal cells Hegde and collaborators showed that VpmaV exhibited the highest adhesion rate of the various Vpmas. Furthermore, they also could show a strong correlation between functions of Vpma proteins in cell adhesion and invasion. This strongly indicates that the variability of these lipoproteins not only causes evasion of the host’s immune response, but also changes the adhesion characteristics of the pathogen to allow it to adapt and spread in different host niches. Interestingly strain GrTh01 reveals two *vpma* loci similar to *M. agalactiae* strain 5632 [44], one located at the *tRNA-lys* gene (locus I) and the other at the 16S rRNA gene (locus II) (Fig. 2). Both loci contain a full-length *xer1* gene but many strongly truncated *vpma* genes respectively residues with locus I having full length copies of *vpmaW*, *vpmaX* and *vpmaZ* and locus II showing solely *vpmaY* in full length. VpmaV, which was shown to have highest adhesion properties to sheep cells, is absent in strain GrTh01. The reduced set of available *vpma* genes and in particular the lack of *vpmaV* could explain the reduced host range and a predilection of the outbreak strain GrTh01 for Caprinae and for the mammary gland as revealed from the clinical and epidemiological data of the outbreak. Our clinical and molecular genetic data on strain GrTh01 show certain correlations to the observations by Chopra-Dewasthaly and collaborators [13] that the various Vpma lipoproteins are involved in host and tissue specificity. However, additional analysis of expressions of Vpma lipoproteins in strain GrTh01 under in vitro and under in vivo conditions would be necessary to confirm these correlations. We assume that the antigenic variation gives *M. agalactiae* the opportunity to infect and invade various hosts and tissues, hence generally causing multiple symptoms in various small ruminants unless certain adhesins are missing thus reducing host range and tissue tropism.

## Data Availability

Full genome sequence data of this work are publicly available from NCBI GenBank https://www.ncbi.nlm.nih.gov/genbank/, a member of the International Nucleotide Sequence Database Collaborations under the accession number CP039447.
